# Supplementary material for: How urban impervious surface shapes bird foraging behavior in an arid city
Source: PeerJ. 2025 Sep 23;13:e19980. doi: 10.7717/peerj.19980 (PMC12466499; doi:10.7717/peerj.19980)
Supplement: Supplemental Information 2 [file peerj-13-19980-s002.docx]

**Supplement**

Supplementary Table 1. List of bird species (common and scientific names) and the frequency with which each was observed as first visitors to popcorn feeding stations in the greater Phoenix metro area, central Arizona, USA. Doves (family: Columbidae) were grouped into native and non-native groups.

| Species Name | Number of Times as First Visitor |
| --- | --- |
| Great-tailed Grackle (*Quiscalus mexicanus*) | 26 |
| Native Doves:  Inca (*Columbina inca*)  White-winged (*Zenaida asiatica*)  Mourning (*Zenaida macroura*) | 8 |
| House Sparrow (*Passer domesticus*) | 7 |
| House Finch (*Haemorhous mexicanus*) | 7 |
| Non-Native Doves:  Eurasian Collared-dove (*Streptopelia decaocto*)  Rock Pigeon (*Columba livia*) | 5 |
| Abert's Towhee (*Pipilo aberti*) | 2 |
| Rock Wren (*Salpinctes obsoletus*) | 2 |
| Cactus Wren (*Campylorhynchus brunneicapillus*) | 1 |
| Northern Mockingbird (*Mimus polyglottos*) | 1 |
| Greater Roadrunner (*Geococcyx californianus*) | 1 |
| Curve-billed Thrasher (*Toxostoma curvirostre*) | 1 |
| European Starling (*Sturnus vulgaris*) | 1 |

Supplementary Table 2. Model selection results of generalized linear mixed models estimating relationships with impervious surface area (ISA) and day-of-year (DOY). ‘Visit or not’ response variables represent whether or not a feeding station was visited by a specific species or guild: Great-tailed Grackle (GTGR), House Finch (HOFI), House Sparrow (HOSP), native doves (NDOV), and introduced doves (IDOV). Relative quality of models was represented using the Akaike Information Criterion adjusted for small sample size (AIC_c_). ‘NA’ values indicate either the inability to calculate the variance explained by modeled fixed effects (R^2^) or failure of models to converge (for AIC_c_).

| **Response Variable** | **Fixed Effect(s)** | **R^2^** | **AIC_c_** |
| --- | --- | --- | --- |
| Visit or not, GTGR | ISA only | 0.026 | 141.933 |
|  | ISA+DOY | 0.031 | 143.693 |
| Visit or not, HOFI | ISA only | 0.269 | 54.424 |
|  | ISA+DOY | 0.253 | 56.476 |
| Visit or not, HOSP | ISA only | 0.376 | 57.592 |
|  | ISA+DOY | 0.376 | 58.463 |
| Visit or not, NDOV | ISA only | 0.236 | 54.750 |
|  | ISA+DOY | 0.229 | 56.853 |
| Visit or not, IDOV | ISA only | 0.002 | 65.727 |
|  | ISA+DOY | 0.022 | 67.421 |
| Latency | ISA only | 0.152 | 887.861 |
|  | ISA+DOY | NA | NA |
| Species Richness | ISA only | NA | 229.437 |
|  | ISA+DOY | NA | 230.673 |


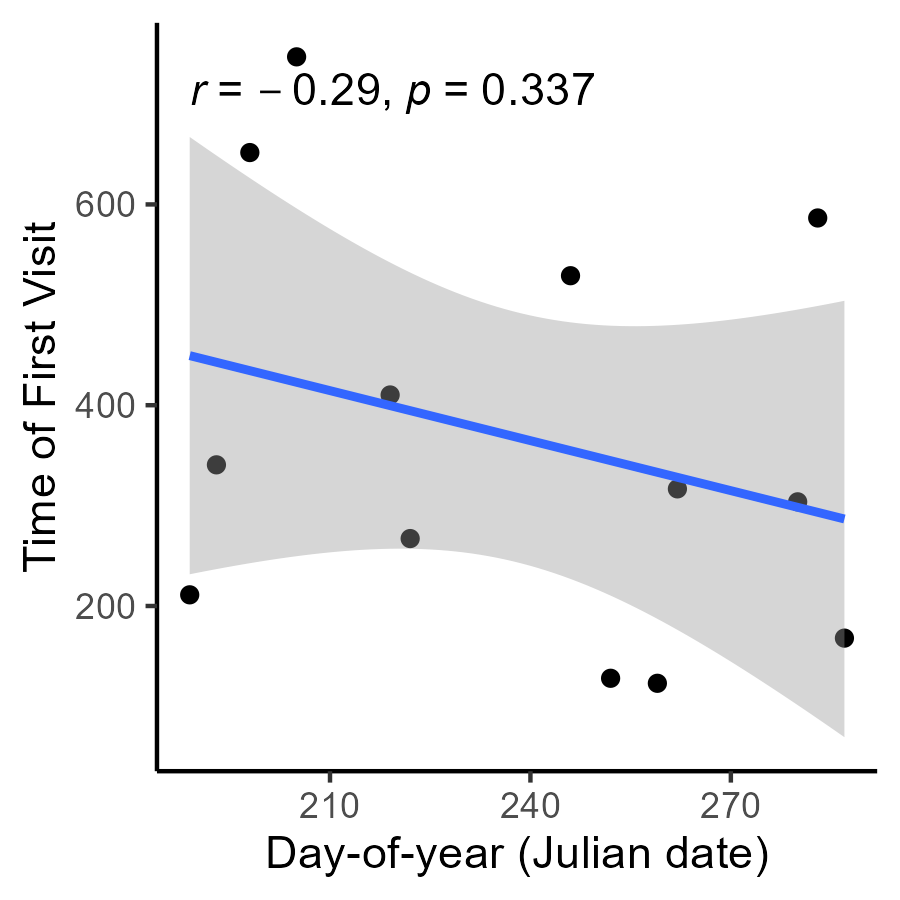


Supplementary Figure 1. Relationship between day-of-year (Julian date of surveys at each site) and time of first visit (mean time until first bird visitation to feeding station).
